# Medetomidine/midazolam/fentanyl narcosis alters cardiac autonomic tone leading to conduction disorders and arrhythmias in mice

**DOI:** 10.1038/s41684-023-01141-0

**Published:** 2023-03-23

**Authors:** Philipp Tomsits, Lina Volz, Ruibing Xia, Aparna Chivukula, Dominik Schüttler, Sebastian Clauß

**Affiliations:** 1 Medizinische Klinik und Poliklinik I, University Hospital Munich, Campus Grosshadern and Innenstadt, Ludwig-Maximilians University Munich (LMU), Munich, Germany; 2 DZHK (German Centre for Cardiovascular Research), Partner Site Munich, Munich Heart Alliance (MHA), Munich, Germany; 3 Institute of Surgical Research at the Walter-Brendel-Centre of Experimental Medicine, University Hospital, LMU Munich, Munich, Germany; 4 Interfaculty Center for Endocrine and Cardiovascular Disease Network Modelling and Clinical Transfer (ICONLMU), LMU Munich, Munich, Germany

**Keywords:** Arrhythmias, Arrhythmias

## Abstract

Arrhythmias are critical contributors to cardiovascular morbidity and mortality. Therapies are mainly symptomatic and often insufficient, emphasizing the need for basic research to unveil the mechanisms underlying arrhythmias and to enable better and ideally causal therapies. In translational approaches, mice are commonly used to study arrhythmia mechanisms in vivo. Experimental electrophysiology studies in mice are performed under anesthesia with medetomidine/midazolam/fentanyl (MMF) and isoflurane/fentanyl (IF) as commonly used regimens. Despite evidence of adverse effects of individual components on cardiac function, few data are available regarding the specific effects of these regimens on cardiac electrophysiology in mice. Here we present a study investigating the effects of MMF and IF narcosis on cardiac electrophysiology in vivo in C57BL/6N wild-type mice. Telemetry transmitters were implanted in a group of mice, which served as controls for baseline parameters without narcosis. In two other groups of mice, electrocardiogram and invasive electrophysiology studies were performed under narcosis (with either MMF or IF). Basic electrocardiogram parameters, heart rate variability parameters, sinus node and atrioventricular node function, and susceptibility to arrhythmias were assessed. Experimental data suggest a remarkable influence of MMF on cardiac electrophysiology compared with IF and awake animals. While IF only moderately reduced heart rate, MMF led to significant bradycardia, spontaneous arrhythmias, heart rate variability alterations as well as sinus and AV node dysfunction, and increased inducibility of ventricular arrhythmias. On the basis of these observed effects, we suggest avoiding MMF in mice, specifically when studying cardiac electrophysiology, but also whenever a regular heartbeat is required for reliable results, such as in heart failure or imaging research.

## Abstract (web summary)

Tomsits and colleagues investigated the effects of medetomidine/midazolam/fentanyl and isoflurane/fentanyl narcosis on murine cardiac autonomic nervous activity and electrophysiology. The results show that, compared with medetomidine/midazolam/fentanyl narcosis, isoflurane/fentanyl narcosis seems to have no effect on cardiac autonomic nervous function and less influence on cardiac electrophysiology.

## Main

Cardiac arrhythmias ranging from harmless premature extra beats to severe disorders such as high-degree atrioventricular (AV) conduction block or ventricular fibrillation affect millions of people worldwide^1–4^. The socioeconomic burden of cardiac arrhythmias is expected to grow with the aging population, given that arrhythmia prevalence increases with age and is already higher than 10% in individuals older than 75 years^5^. Current treatment options depend on the specific arrhythmia and include antiarrhythmic drugs and catheter ablation; however, these treatments are often insufficient and accompanied by serious side effects^6,7^. The detailed mechanisms of arrhythmogenesis are still incompletely understood, hindering the development of targeted therapies^8^. Basic research is therefore critical to provide new insights into the pathophysiology of arrhythmias and to allow the development of new therapies^9^.

Choosing an appropriate preclinical arrhythmia model is a complex and multifactorial decision^8,10^. Investigators generally follow a stepwise translational approach: initial fundamental investigations are performed in vitro to identify target structures; these studies are followed first by in vivo investigations using small animals such as mice to confirm initial findings; then more extensive in vivo studies are done in larger animals such as rabbits or pigs to validate the results and to perform preclinical testing before finally first in-human clinical trials can be conducted. Mouse models have an essential role in this translational road, because they can conveniently be genetically modified, have a short generation time and can be housed and bred in most institutions at reasonable costs^8^. Because of these advantages, mouse models are widely used for initial studies on cardiac electrophysiology (EP) in vivo. While assessment of basic electrocardiogram (ECG)-based parameters can be achieved noninvasively, a comprehensive in vivo assessment of parameters such as sinus node or AV node function can only be realized invasively^11^. Regardless of the investigated species, this invasive assessment comes at the cost of a potential confounder: the need for anesthetics. This requirement is based on animal welfare considerations supported by governmental regulations as well as the need for a controlled environment to introduce a catheter into the heart. However, results obtained under the influence of anesthetics may differ from those obtained in conscious animals, because of direct or indirect effects of anesthetics on cardiac EP^12^, which may represent a major potential confounder.

In the past decades, various narcosis regimens have been proposed and successfully established for rodents^13,14^. The combinations of medetomidine, midazolam and fentanyl (MMF)^13,15,16^ as well as isoflurane and fentanyl (IF) are among the most popular regimens for in vivo cardiovascular studies^17^. Utilization of medetomidine has been reported to cause cardiocirculatory depression in small and large animals, ranging from bradycardia up to third-degree AV block and even arrhythmia^16,18–24^. Although these effects seem to be dose dependent, even the smallest doses (1 and 2 µg/kg), which are 200–5,000 times below the recommended doses for murine application (0.2–50.0 mg/kg (refs. ^25,26^)), show cardiocirculatory depression in a dose titration study in dogs^27^. Midazolam as well can show cardiodepressant effects and even alter calcium handling in cultured rat ventricular cardiomyocytes^28^. On the other hand, regimens based on isoflurane have been used in a broad range of species^29–31^ and have been demonstrated to maintain physiological cardiovascular parameters and EP in humans as well as guinea pigs^32,33^. Although various studies have investigated the general cardiovascular effects and the effects on surface ECG of different narcosis regimens in mice, only a single study by Appleton et al.^34^ has invasively evaluated the effects of narcosis on EP in mice so far. The study focused on pentobarbital, and a combination of ketamine, xylazine and acepromazine, which to our knowledge are both not commonly used in murine electrophysiological studies. Given that even studies assessing cardiac EP noninvasively have shown the fundamental impact of different narcosis regimens on ECG^35,36^ and given the increasing importance of transgenic mouse models in arrythmia research, a systematic assessment of narcosis-related effects in general, and specifically of MMF and IF, on cardiac EP in mice is highly needed.

In this Article, we hypothesized that narcosis-induced cardiodepression leads to significant alteration of electrophysiological properties of the heart. To test this hypothesis, we investigated the influence of MMF and IF narcosis on murine cardiac autonomic nervous activity and EP and evaluated the applicability of these regimens for arrhythmia research in mice.

## Results

### Experimental setup

**Fig. 1:**
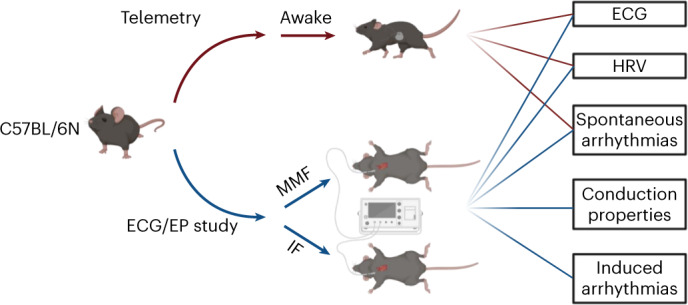
Study design. A total of *n* = 29 C57BL/6N mice were randomly assigned to telemetry (without narcosis) or ECG/invasive EP study with MMF or IF narcosis. Basic ECG parameters, HRV parameters, sinus node and AV node function, refractory periods, susceptibility to arrhythmias and spontaneous arrhythmias were assessed. Figure created with BioRender.com.

A total of 29 C57BL/6N mice were randomly assigned to either telemetry (without narcosis) or ECG/invasive EP study with MMF or IF narcosis (Fig. 1). Telemetry transmitters were implanted in seven mice (four female/three male), which served as controls for baseline ECG parameters without narcosis. ECG and invasive EP studies were performed in 22 mice under narcosis: *n* = 10 for MMF (five female/five male); *n* = 12 for IF (five female/seven male). Basic ECG parameters, spontaneous arrhythmias and conduction disorders, heart rate variability (HRV) parameters, sinus node and AV node function, refractory periods and atrial and ventricular susceptibility to arrhythmias were assessed.

### Narcosis influences heart rate and cardiac conduction

**Fig. 2:**
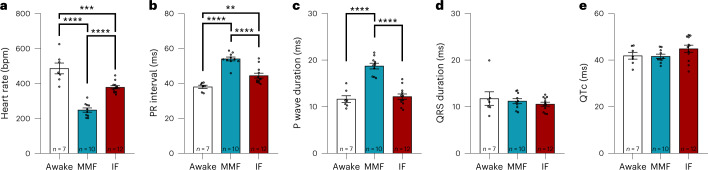
ECG parameters. **a**–**e**, ECG measurements obtained from awake mice were compared with those obtained under MMF or IF narcosis. **a**, Heart rate. **b**, PR interval. **c**, P wave duration. **d**, QRS duration. **e**, QTc (calculation was done according to Mitchell^60^). *n* = 7 for awake, *n* = 10 for MMF and *n* = 12 for IF, values presented as mean ± s.e.m.*P < 0.05, **P < 0.01, ****P < 0.0001, using one-way analysis of variance with Tukey’s multiple comparison test.

To investigate the effects of narcosis on surface ECG, we compared measurements obtained from awake animals to those obtained under MMF or IF narcosis (Fig. 2; mean ± standard error of the mean (s.e.m.), representative traces can be found in Supplementary Fig. 1). Both narcosis regimens significantly lowered mean heart rate (Fig. 2a), but while this effect can be considered moderate in the IF group, severe bradycardia resulted from narcosis with MMF (awake mice, 486.00 ± 30.82 bpm; MMF, 248.20 ± 12.38 bpm; IF, 378.60 ± 9.11 bpm). The PR interval was significantly prolonged under narcosis (Fig. 2b), with a pronounced prolongation in the MMF group (awake, 38.05 ± 0.95 ms; MMF, 54.01 ± 1.12 ms; IF, 44.43 ± 1.38 ms), suggesting slowed AV conduction^37^. MMF also significantly increased P wave duration versus both awake mice and IF group (Fig. 2c), indicating slowed atrial conduction (awake, 11.65 ± 0.72 ms; MMF, 18.74 ± 0.61 ms; IF, 12.16 ± 0.57 ms). Ventricular conduction and repolarization remained unaffected by MMF and IF (Fig. 2d,e).

### MMF alters HRV parameters

**Fig. 3:**
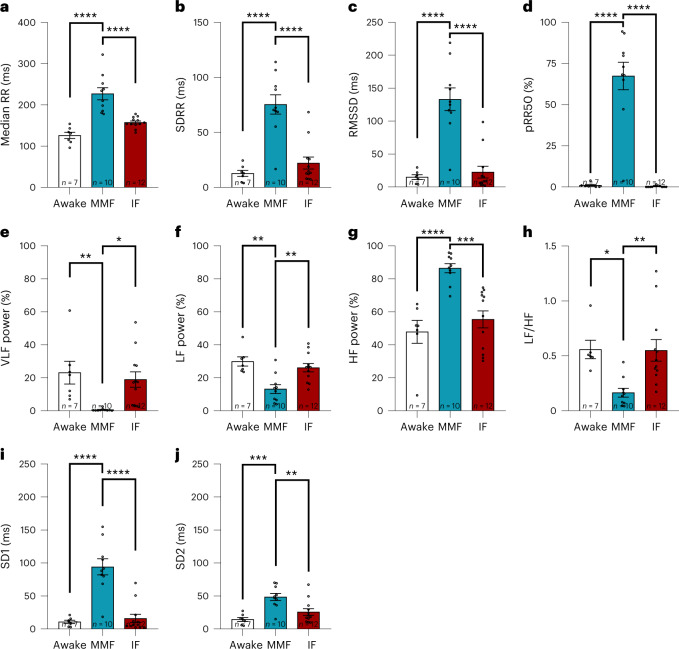
HRV analysis. **a**–**j**, HRV parameters obtained from awake mice were compared with those obtained under MMF or IF narcosis. **a**, Median RR interval. **b**, SDRR. **c**, RMSSD. **d**, pRR50. **e**, Percentage of the VLF oscillation (0.00–0.15 Hz). **f**, Percentage of the LF oscillation (0.15–1.50 Hz). **g**, Percentage of the HF oscillation (1.5–5.0 Hz). **h**, LF/HF. **i**, SD1. **j**, SD2. *n* = 7 for awake, *n* = 10 for MMF and *n* = 12 for IF, values presented as mean ± s.e.m.*P < 0.05, **P < 0.01, ***P < 0.001, ****P < 0.0001, using one-way analysis of variance with Tukey’s multiple comparison test.

HRV parameters (Fig. 3; mean ± s.e.m.) provide insight into the influence of autonomic nervous innervation on the heart by beat-to-beat analysis of changes in RR intervals^38^. Time domain (Fig. 3b–d), frequency domain (Fig. 3e–h, representative plots can be found in Supplementary Fig. 3) and nonlinear analysis (Fig. 3i,j, representative plots can be found in Supplementary Fig. 2) reflect different aspects of the autonomous nervous tone^39^. While IF narcosis had no influence on any HRV parameter when compared with awake mice, MMF narcosis significantly increased median RR (Fig. 3a; awake, 126.10 ± 7.62 ms; MMF, 227.30 ± 14.62 ms; IF, 157.50 ± 3.77 ms); time domain analysis also revealed higher standard deviation of all RR intervals (SDRR, Fig. 3b; awake, 12.72 ± 2.76 ms; MMF, 75.46 ± 8.75 ms; IF, 22.10 ± 5.45 ms), higher square root of the mean of the sum of the squares of differences between adjacent RR intervals (RMSSD, Fig. 3c; awake, 15.12 ± 3.51 ms; MMF, 133.10 ± 17.14 ms; IF, 22.59 ± 8.88 ms) and a higher percentage of differences between adjacent RR intervals that are greater than 50 ms (pRR50, Fig. 3d; awake, 1.01 ± 0.50%; MMF, 67.37 ± 8.33%; IF, 0.37 ± 0.13%) in MMF mice compared with awake mice, suggesting a predominant parasympathetic tone. Frequency domain analysis showed reduced very low frequency (VLF, Fig. 3e; awake, 23.13 ± 6.94%; MMF, 0.59 ± 0.24%; IF, 18.97 ± 4.69%) and low frequency (LF) with MMF narcosis versus awake mice (Fig. 3f; awake, 29.89 ± 2.80%; MMF, 13.19 ± 2.71%; IF, 26.13 ± 2.52%), suggesting reduced baroreflex sensitivity^38^. Frequency domain analysis also showed increased high frequency (HF) with MMF (Fig. 3g; awake, 47.86 ± 6.90%; MMF, 86.48 ± 2.75%; IF, 55.43 ± 5.15%), supporting the time domain results suggesting predominant parasympathetic activity. These conclusions are also supported by the reduced rate of LF to HF quotient (LF/HF) in MMF narcosis (Fig. 3h; awake, 0.56 ± 0.08; MMF, 0.16 ± 0.04; IF, 0.55 ± 0.10). Nonlinear analysis showed increased overall variability for MMF narcosis as reflected by increased Poincaré standard deviation 1 (SD1, Fig. 3i; awake, 10.70 ± 2.48 ms; MMF, 94.14 ± 12.13 ms; IF, 15.98 ± 6.28 ms) and Poincaré standard deviation 2 (SD2, Fig. 3j; awake, 14.43 ± 3.04 ms; MMF, 48.45 ± 5.34 ms; IF, 25.73 ± 5.04 ms) compared with awake and freely moving mice.

### MMF impairs sinus node and AV node function

**Fig. 4:**
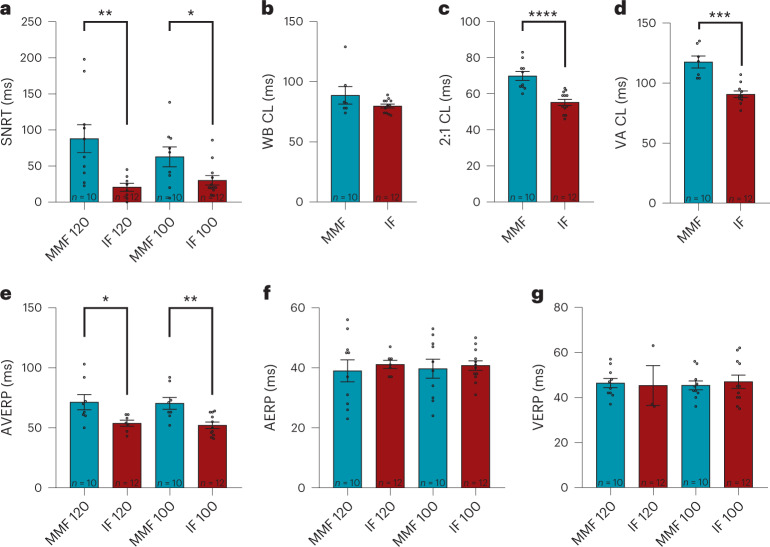
EP study. **a**–**g**, EP study was performed in mice under MMF or IF narcosis. **a**, SNRT, corrected by subtraction of the individual atrial basic cycle length. **b**, WB CL. **c**, 2:1 CL. **d**, VA CL. **e**, AVERP. **f**, AERP. **g**, VERP. *n* = 10 for MMF and *n* = 12 for IF, values presented as mean ± s.e.m. *P < 0.05, **P < 0.01, ***P < 0.001, ****P < 0.0001 Mann–Whitney test.

Invasive assessment of sinus node function by EP study revealed a significant prolongation of the sinus node recovery time (SNRT) with MMF compared with IF narcosis indicating sinus node dysfunction (Fig. 4a; mean ± s.e.m.; SNRT at 120 ms drive train: MMF, 87.93 ± 19.39 ms; IF, 20.63 ± 5.52 ms; at 100 ms drive train: MMF, 62.76 ± 13.72 ms; IF, 30.13 ± 6.58 ms).

To further investigate AV conduction properties, Wenckebach cycle length (WB CL), 2:1 AV conduction cycle length, retrograde conduction cycle length and the effective refractory period of the AV node were assessed (Fig. 4b–e; mean ± s.e.m.). These parameters further demonstrated a severe AV node dysfunction under the influence of MMF: whereas WB CL (Fig. 4b; MMF, 88.71 ± 7.26 ms; IF, 79.83 ± 1.64 ms) showed only a trend towards a prolongation, 2:1 conduction cycle length (2:1 CL, Fig. 4c; MMF, 69.90 ± 2.47 ms, IF, 55.25 ± 1.72 ms) as well as retrograde conduction cycle length (VA CL, Fig. 4d; MMF, 117.60 ± 4.98 ms; IF 90.70 ± 2.76 ms) and AV effective refractory period (AVERP, Fig. 4e; at 120 ms drive train: MMF, 71.38 ± 6.26 ms; IF, 53.86 ± 2.60 ms; at 100 ms drive train; MMF, 70.38 ± 4.96 ms; IF, 52.18 ± 2.69 ms) were significantly increased with MMF compared with IF narcosis. The atrial effective refractory period (AERP) as well as the ventricular effective refractory period (VERP) did not differ between groups (as seen in Fig. 4f; mean ± s.e.m.; AERP at 120 ms drive train: MMF, 39.00 ± 3.68 ms; IF, 41.14 ± 1.35 ms; AERP at 100 ms drive train: MMF, 39.70 ± 3.20 ms; IF, 40.75 ± 1.58 ms; and Fig. 4g; mean ± SEM; VERP at 120 ms drive train: MMF, 46.40 ± 2.03 ms; IF 45.33 ± 8.84 ms; VERP at 100 ms drive train: MMF, 45.40 ± 1.98 ms; IF, 47.00 ± 2.96 ms).

### MMF enhances arrhythmogenesis

**Fig. 5:**
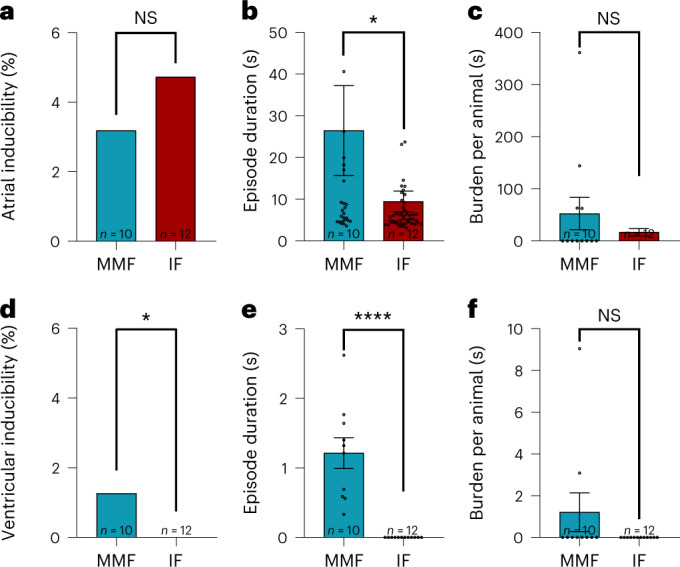
Arrhythmia inducibility and quantification. **a**–**f**, Arrhythmogenesis was assessed in mice under MMF or IF narcosis**. a**, Atrial inducibility. **b**, Atrial mean episode duration. **c**, Mean burden of atrial arrhythmia per animal. **d**, Ventricular inducibility. **e**, Ventricular mean episode duration. **f**, Mean burden of ventricular arrhythmia per animal. NS, not significant. *n* = 10 for MMF and *n* = 12 for IF, values are presented as mean ± s.e.m. *P < 0.05 chi-square test (for **d**). *P < 0.05 Mann–Whitney test (for **b**), ****P < 0.0001 unpaired Student’s *t*-test (for **e**).

Atrial arrhythmia inducibility by programmed electrical stimulation and burst stimulation did not differ between narcosis regimens (Fig. 5a; MMF, 3.18%; IF 4.72%). Nevertheless, animals anesthetized with MMF showed a statistically significant increased duration of atrial arrhythmia episodes (Fig. 5b; mean ± s.e.m.; MMF, 26.47 ± 10.78 s; IF 9.47 ± 2.49 s), a trend towards an increased arrhythmia burden per animal (Fig. 5c; mean ± s.e.m.; MMF, 63.06 ± 36.39 s; IF 16.74 ± 7.14 s) and a higher total arrhythmia burden (Supplementary Fig. 4a; MMF, 741.20 s; IF, 457.58 s) compared with IF narcosis.

While no ventricular arrhythmia could be induced with IF narcosis, animals under the influence of MMF showed significant inducibility (Fig. 5d; MMF, 1,26%; IF, 0%), with consequently higher mean episode duration (Fig. 5e; mean ± s.e.m.; MMF, 1.21 ± 0.22 s; IF, 0 s), and a trend towards a higher burden per animal (Fig. 5f; mean ± s.e.m.; MMF, 1.21 ± 0.92 s; IF, 0 s) as well as higher total arrhythmia burden (Supplementary Fig. 4b; MMF, 12.13 s; IF, 0 s).

**Fig. 6:**
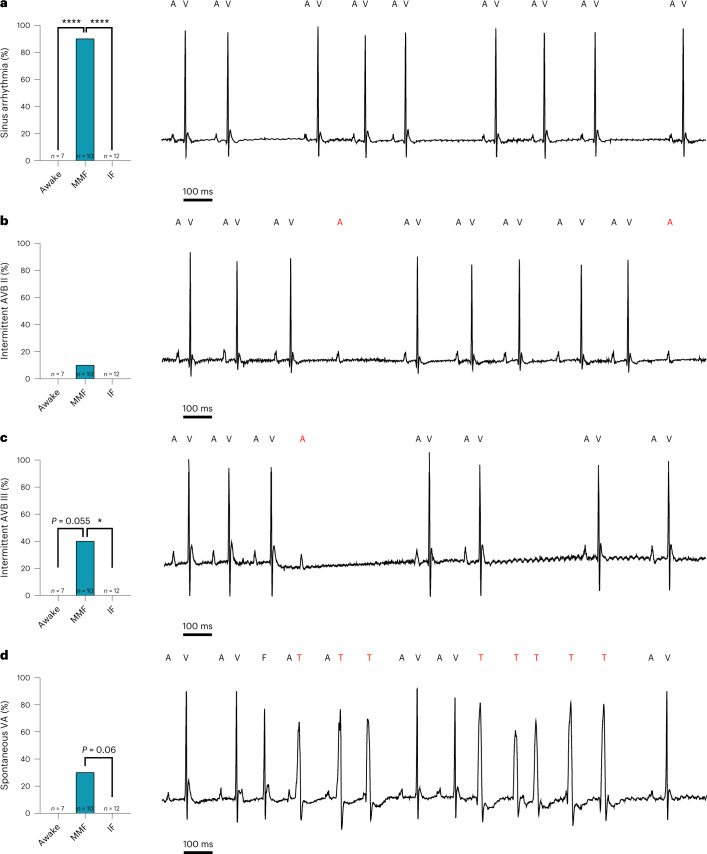
Spontaneous arrhythmias. **a**–**d**, Spontaneous occurrence of arrhythmia was analyzed from ECGs of awake mice and mice under MMF or IF narcosis. **a**, Quantification of sinus arrhythmia calculated as the percentage of animals per group showing the occurrence in the surface ECG (left) and a corresponding representative trace from MMF group (right). **b**, Quantification of intermittent AV block (AVB) type II Wenckebach (left) calculated as the percentage of animals per group showing the occurrence and a corresponding representative trace from MMF group (right). **c**, Quantification of intermittent AV block III (left) calculated as the percentage of animals per group showing the occurrence and a corresponding representative trace from MMF group (right). **d**, Quantification of abnormal spontaneous ventricular activity (left) calculated as the percentage of animals per group showing the occurrence and a corresponding representative trace from MMF group (right). Red, non-conducted atrial beats and spontaneous ventricular activity. A, atrial beat; F, fusion beat; T, spontaneous ventricular activity; V, ventricular beat. *n* = 7 for awake, *n* = 10 for MMF and *n* = 12 for IF, *P < 0.05, ****P < 0.001 chi-square test.

ECG assessment revealed spontaneous occurrence of sinus arrhythmia (Fig. 6a; 9/10 animals), high-grade AV block (Fig. 6b, c; 5/10 animals) and nonsustained abnormal spontaneous ventricular activity (Fig. 6d; 3/10 animals) with MMF narcosis, whereas no such disorders occurred in awake mice or those undergoing IF narcosis.

## Discussion

Mouse models are widely used to study cardiovascular (patho)physiology. Increasing EP studies have been performed in mice within the past years to characterize their cardiac electrical phenotype. While clinical EP studies are usually performed in conscious patients, animal studies require anesthetics, which potentially affect cardiac EP and arrhythmogenesis. Thus, establishing a narcosis regimen with minimal impact on EP is essential to obtain valid results. Currently, MMF and IF are among the most popular anesthesia regimens to invasively study EP in mice, but a direct comparison of their effects on autonomous nervous function as well as on cardiac EP is lacking so far.

An ideal anesthesia would have no side effects on cardiac EP itself. To be able to address the influence of narcosis regimens on cardiac EP, we used animals equipped with implantable telemetry devices to record ECG parameters and spontaneous arrhythmias in conscious mice. Data from these animals were then compared with data obtained from mice under MMF and IF narcosis. Telemetry data were obtained in a lead II position around noon, and ECG data during narcosis in a lead I position at full onset of narcosis during daytime. There is a lack of experimental data on inter-lead variability in ECG intervals; although inter-lead variability may have contributed to differences seen between controls and IF/MMF animals, unchanged QRS or QTc duration between the three groups suggests otherwise. As circadian effects can influence parameters such as heart rate, which in median is lower during light cycles^40^, we compared telemetry data obtained around noon to ECGs obtained under anesthesia during daytime from 10:00 to 18:00 within the 12 h light cycle (7:00 to 19:00).

As reported by prior studies^16,19,20,30,31^, both regimens lowered heart rate compared with awake and freely moving animals. This effect was significantly less pronounced in mice treated with IF, which is well in line with previous findings showing an almost preserved heart function under isoflurane narcosis^15,29,41^ in various species. Both protocols prolonged the PR interval, with only a slight increase under IF and a profound increase under MMF narcosis. This indicates, together with a significantly prolonged P wave duration, that especially MMF narcosis has a profound effect on both atrial and AV conduction. Neither of the protocols changed QRS duration or QTc interval, suggesting no direct influence on ventricular conduction or repolarization. Besides severe bradycardia, almost all animals treated with MMF developed sinus arrhythmia and some showed higher-grade AV block and even spontaneous ventricular activity as previously described for dogs^20^, horses^18,19^, rats^24^ and a Sumatran tiger^23^ treated with medetomidine.

Previous studies have suggested that conduction impairments are caused by the alpha 2-adrenoceptor agonist^42^ medetomidine, although the underlying mechanism is not completely clear. It is known that medetomidine induces a decrease in the central release of norepinephrine^42^, suggesting that the effects of medetomidine could be mediated by its influence on the autonomous innervation of the heart, tilting the scale towards a predominant vagal tone. This hypothesis is in line with our data clearly showing in MMF mice a remarkable increase in SDRR, RMSSD and pRR50, which are considered valid indicators of an increased vagal tone^38^. Frequency domain analysis also showed a distinct effect of MMF narcosis. Vagal tone correlates with the HF component of short-term HRV analysis^38^, which in our study was increased among animals receiving MMF, suggesting an increased vagal tone in this group. SD1 represents short-term variability of the NN intervals and is mainly influenced by parasympathetic activity^43^. The significantly higher SD1 in mice treated with MMF further hints towards higher parasympathetic activity in this group. SD2 is said to correlate more with sympathetic activity^44^, and individuals with diabetes for instance show a phenotype with reduced SD1 and increased compensatory SD2 (ref. ^44^). The higher SD2 values in the MMF group could suggest a sympathetic reaction to the overly activated parasympathetic system, but further studies are clearly warranted to unveil the exact underlying mechanisms. Of note, sinus arrhythmia, among other factors, can influence the accuracy of HF analysis by causing heart rhythm fragmentation^45^. As sinus arrhythmia is very much present in MMF-treated mice, it remains unclear whether the observed alterations in HRV are solely due to direct effects of MMF on the autonomic nervous tone. However, it is important to emphasize that IF narcosis did not have any effects on HRV analysis in our study; taken together with the previous findings discussed above, it can therefore be assumed that IF narcosis has little to no effects on cardiac autonomic nervous tone. Nevertheless, further studies using approaches such as pharmacological interventions are needed to clarify if the effects of MMF observed here are directly caused by vagal modulation.

Our invasive EP studies have provided further evidence of adverse effects of MMF narcosis. Given that surface ECG measurements demonstrated little to no effect of IF narcosis on cardiac EP, it can be assumed that the prolonged SNRT observed in MMF-treated animals is indeed a prolongation in this group rather than a shortening under the influence of IF. This finding is also in line with findings from young patients undergoing EP studies under isoflurane, showing no effect of narcosis on sinus node function and AV node function^33^. The same inference can be made for the impaired AV node function under MMF narcosis (as indicated by an increased 2:1 CL, an impaired retrograde AV node conduction as well as an increased AVERP), which is further supported by the spontaneous occurrence of sinus arrhythmia and higher-grade AV block in the MMF group. It is striking that sinus node and AV node function are affected in such a strong fashion by MMF narcosis, whereas ventricular conduction remains unchanged. Both effects could be mediated by an increase in vagal tone, as demonstrated by previous studies^46^. The spatial distribution of autonomic innervation of the heart further supports this hypothesis, as sympathetic as well as parasympathetic fibers mainly innervate sinus and AV node^47,48^; therefore, the effects observed in our study could be explained by the effects of MMF on the autonomic balance. By contrast, the ventricular myocardium is only sparsely innervated by efferent parasympathetic fibers.

Atrial arrhythmia induction by programmed electrical stimulation and burst stimulation showed no difference between MMF and IF groups. Interestingly, in MMF-treated mice episodes were significantly longer and arrhythmia burden, both per individual animal and in total, was increased without reaching statistical significance. Whether this is due to MMF enhancing the susceptibility or to IF inhibiting arrhythmias is unclear; however, previous studies suggest protective effects of isoflurane^49,50^, potentially caused by a prolonged action potential duration under 1.0–1.5% vol/vol isoflurane^32^. These findings are further supported by the reduced susceptibility to ventricular arrhythmias in mice under IF in our study.

Overall, this study clearly shows a general impact of narcosis on cardiac EP in mice. While this effect is profound for MMF narcosis, causing conduction disorders and ultimately arrhythmias, IF narcosis exerts only little effects on EP and no effects on cardiac autonomous nervous regulation, as depicted by HRV analysis. MMF as well as IF resulted in rapid onset and sufficient anesthesia, making animal handling convenient. Nevertheless, considering the intraperitoneal (i.p.) bolus concept of MMF narcosis, optimal narcosis depth was harder to achieve, and experiments had to be stopped for further injection after 45 min. Isoflurane allows for a better control of the anesthesia depth since its dose can easily be adjusted according to the animal’s heart rate. Whether these findings translate to larger mammals and ultimately humans remains speculative for the moment. Given that the i.p. bolus concept of murine MMF narcosis causes higher peaks in drug concentrations compared with continuous intravenous (i.v.) administration, which is commonly used in larger mammals, a less profound effect on cardiovascular function and EP may be expected with i.v. administration of the regimens. However, a growing body of evidence suggests that anesthetics such as medetomidine can cause similar arrhythmic effects in large mammals also under continuous i.v. application^18^. Further studies are necessary to determine if medetomidine should especially be avoided in individuals prone to arrhythmia. As this study is based on final experiments, potential reversibility and time course of the observed MMF and IF effects could not be assessed. However, numerous studies have used both regimens for survival surgeries and there is no evidence of irreversible effects, suggesting that the effects on cardiac EP may be reversible as well. Future survival studies, in which EP is assessed by esophageal catheter, for example, are warranted to investigate reversibility of the observed effects.

Besides the two narcosis regimens studied in this manuscript, there are various other possible forms of anesthesia, including isoflurane-based regimens combined with other opioids than fentanyl or barbiturates, as well as regimens based on injectables such as ketamine/xylazine, or avertin and pentobarbital^51,52^. Some of these protocols are more widely used for studies in mice in other contexts than arrhythmia^53^. Whether certain other anesthesia protocols may lead to less interference with murine cardiac EP remains elusive. However, available data on parameters such as hemodynamics and heart rate^34–36,53–55^ suggest that all protocols have the potential to largely interfere with cardiac function and EP; therefore, further invasive studies are warranted to compare IF regimen with other isoflurane-based or injectable protocols, and identify the best compromise for invasive arrhythmia research in rodents.

For a long time, females have been excluded from clinical and basic research, on the assumption that the test results obtained from male individuals would equally apply to females^56^. In recent years, multiple reports have suggested that findings from male populations may not always be applicable to female cohorts^57,58^, and have thus emphasized the need to consider sex as a major variable in all experimental study designs^59^. Consequently, this study included C57BL/6N mice of both sexes distributed equally among groups. However, the small group sizes limit robust sex-specific analysis and we cannot exclude that some of the observed effects on EP may be more or less pronounced depending on the sex of the animal. As it is becoming clear that sex is an important biological variable affecting experimental outcomes, further studies are needed to shed light on the sex-specific effects of drugs on cardiac EP in mice, but also in lager mammals and ultimately in humans.

In summary, this study shows that, compared with MMF narcosis, IF narcosis is easier to control, seems to have no effect on cardiac autonomic nervous function and does interfere far less with cardiac EP. To comply with the 3R concept (replacement, reduction and refinement), identifying a suitable narcosis regimen for arrhythmia research is essential. Reducing confounders limits variance in results, increases statistical power and allows for smaller cohorts (reduction). Decreasing the adverse effects of mandatory anesthesia further increases animal welfare (refinement). Ultimately, if more research can be reliably performed on small animals (for example, for screening for novel drug candidates), a lower number of large animal models will be needed on the translational road to clinical medicine (replacement). Further studies are warranted to compare IF with other narcosis regimens, but based on the results of this study and the above mentioned considerations, we strongly suggest avoiding MMF regimen in favor of IF narcosis for EP and arrhythmia studies in mice. Furthermore, these considerations also apply to other fields of research such as heart failure or imaging studies, given that an irregular heartbeat may affect contractile function and prevent triggered computed tomography/magnetic resonance scans.

## Methods

All experimental procedures were approved and performed in accordance with the regulations by the Regierung von Oberbayern (ROB-55.2-2532.Vet_02-19-86 and ROB-55.2-2532.Vet_02-16-106), the ARRIVE guidelines^61^ for the reporting of in vivo experiments in animal research and the Guide for the Care and Use of Laboratory Animals^62^.

### Animals

Altogether, 29 female and male C57BL/6N mice (body weight 21.4–30.4 g; age: 8–10 weeks (4 female/3 male for telemetry, 5 female/7 male for IF and 5 female/5 male for MMF narcosis)) from our own breeding were used. Animals were housed in numbers of 4 to 5 per cage in our animal facility under temperature (24 ± 1 °C) and light (12:12 light–dark cycle, light on from 7:00) control. Standard laboratory mouse chow and tap water were available ad libitum.

### Study design

The goal of this study was to determine whether the choice of the narcosis regimen has an impact on cardiac EP and arrhythmogenesis in mice. To address this issue, we studied the effects of two of the most commonly used narcosis regimens for mice in arrhythmia research (MMF and IF) on ECG and HRV parameters using awake and freely moving animals as controls. Further, we invasively assessed sinus node function, AV node function as well as electrophysiological properties of atrial and ventricular tissues in vivo under the influence of MMF or IF narcosis.

### Telemetry

Continuous ECG telemetry was performed by implanting ETA-F10 transmitters (Data Science International) under general anesthesia as previously described^63,64^. Transmitters were implanted in the abdomen, and the leads were tunneled subcutaneously to the upper right and lower left chest, resulting in a lead II position. Telemetry data were recorded continuously via a receiver (RPC-1 Data Science International) placed under the mouse cage. Data analysis was performed using LabChart Pro software. Five-minute recordings around noon with good signal-to-noise ratio were averaged to obtain baseline ECG parameters and were used to analyze HRV. The same 5-min sections were visually screened for arrhythmias by two experienced cardiologists.

### Narcosis

#### IF

Narcosis was induced by administration of 2–3% vol/vol isoflurane driven by 95% oxygen (1 l/min), using an induction chamber (Hugo Sachs Elektronik). Once narcosis was established, the animal was moved to a surgical platform (Kent Scientific) and fixed in a supine position on a surgical suite (Kent Scientific) equipped with a temperature control module (Kent Scientific) to continuously keep temperature at 37 °C throughout the entire protocol guided by a rectal probe. We used a vaporizer (Hugo Sachs Elektronik) for precise regulation of inspiratory isoflurane concentration and maintained narcosis with 1.5% vol/vol isoflurane. Intraperitoneally, 0.05 mg/kg body weight fentanyl was applied, and toe pinch reflex was checked to assess anesthesia. Once toe pinch reflex was negative, we proceeded with the experiments.

#### MMF

Narcosis was induced by i.p. injection of medetomidine 0.5 mg/kg body weight, midazolam 5 mg/kg body weight and fentanyl 0.005 mg/kg body weight. Once narcosis was established, the animal was moved to a surgical platform (Kent Scientific) and fixed in a supine position on a heat platform to control temperature at 37 °C guided by a rectal probe. Toe pinch reflex was checked and once negative, we proceeded with the experiments. If needed, an additional half-induction dose was administered intraperitoneally to maintain narcosis 45 min after onset.

### ECG

Three needle electrodes (29 G, AD Instruments) were subcutaneously inserted to allow a lead I position, and data were obtained using an amplifier (AD Instruments), Powerlab (AD Instruments) and LabChart Pro (AD Instruments). Data analysis was performed using LabChart Pro software. Five-minute recordings were obtained right after complete narcosis onset and after the animal was placed on the surgical pad, averaged to obtain ECG parameters and used to analyze HRV in LabChart. Experiments took place during daytime from 10:00 to 18:00. Time domain parameters are defined as follows: average RR, mean RR interval; median RR, median RR interval; SDRR, standard deviation of the RR interval; SDSD, standard deviation of successive RR interval differences between adjacent RR intervals; RMSSD, root mean square of successive RR interval differences; and pRR50, percentage of successive RR interval differences longer than 50 ms. The HRV Module uses the Lomb Periodogram nonparametric method for spectral analysis, which computes the spectral frequencies directly from the unevenly sampled tachogram. For frequency domain analysis, VLF band was between 0.00 and 0.15 Hz; LF band between 0.15 and 1.50 Hz and HF band between 1.5 and 5.0 Hz, as predefined by LabChart for mice. The Poincaré analysis plots RR interval (RR_*n*_) against the subsequent RR interval (RR_*n*+1_). The RR interval time series is defined as RR_*n*_, where *n* = 1… *N*. SD1 and SD2 are second moment measures of the distribution of points around the line of identity (RR_*n*_ = RR_*n*+1_), and characterize the Poincaré plot. For a detailed description of the complex mathematics behind this automated module, the interested reader may be referred to the manufacturer’s manual, which can be found in the ‘Help’ section of the LabChart software. Sinus arrhythmia (defined as visibly irregular sinus rhythm), AV block (°IIA, increasing PR interval until AV conduction block; (intermittent) °III, (intermittent) AV conduction block) and abnormal spontaneous ventricular activity (defined as 4 or more consecutive spontaneous ventricular beats) were analyzed visually from these 5-min ECGs by two experienced cardiologists.

### EP study

After disinfection of the right collar region, a short incision was made along the sternocleidomastoid muscle with subsequent dissection of the internal jugular vein. Through an incision, an octopolar 1.1F EP catheter (Millar Inc.) was inserted into the vein and the tip was carefully placed into the right ventricle under ECG guidance. Positioning was correct when the distal electrodes showed a ventricular signal, and the proximal electrodes showed an atrial signal. Data were recorded using the same hardware as for the ECG and a stimulator (ISO-STIM-01D NPI electronic) was used to generate electrical impulses. EP study was performed as previously described^64–66^. In brief, programmed electrical stimulation followed a standard protocol with 120 ms and 100 ms drive trains and single extra stimuli to measure function of the AV node and the conduction properties of atrial and ventricular tissue. The WB CL was measured by progressively faster atrial pacing rates. Retrograde (VA) conduction cycle length was measured by progressively slower ventricular pacing rates. Sinus node function was determined by measuring the SNRT following 30 s of pacing at two cycle lengths (120, 100 ms) and corrected by subtraction of the corresponding basic cycle length. A S1/S2 protocol was used to assess atrial and ventricular refractory periods at 120 and 100 ms basic cycle length, respectively. Arrhythmia induction consisted of programmed electrical stimulation in right atrium and right ventricle using double (S1/S2/S3) extra stimuli of 40–20 ms with 5 ms decrements at 120 and 100 ms basic cycle length. Burst pacing was performed with application of two 3 s and 6 s bursts at rates of 40–10 ms in 5 ms decrements. Atrial arrhythmia was defined as high frequent polymorphic atrial excitation with regular/irregular ventricular conduction or a clear change in atrial basic cycle length post stimulation with morphologic change of the atrial signal indicating ectopic origin, either of them lasting for ≥3 s. Ventricular arrhythmia after programmed stimulation was defined as occurrence of four or more consecutive ventricular beats after stimulation. Inducibility was calculated as the number of successful stimulation maneuvers over all stimulation maneuvers, and burden represents the sum of all respective episodes of one animal or an entire group (IF/MMF).

### Statistical analysis

All values are presented as mean ± s.e.m. All statistical analyses were conducted with GraphPad Prism software version 8.0.1. Data were tested for normality using the D’Agostino–Pearson normality test. Statistical significance was assessed by two-sided Student’s *t*-test for normally distributed data. If normal distribution assumption was not valid, statistical significance was evaluated using the two-sided Mann–Whitney test. For multiple comparisons, one-way analysis of variance with Tukey’s multiple comparison test was used. Arrhythmia inducibility was analyzed by chi-square test. A *P* value <0.05 was considered as statistically significant.

### Reporting summary

Further information on research design is available in the Nature Portfolio Reporting Summary linked to this article.

## Online content

Any methods, additional references, Nature Portfolio reporting summaries, source data, extended data, supplementary information, acknowledgements, peer review information; details of author contributions and competing interests; and statements of data and code availability are available at 10.1038/s41684-023-01141-0.

## Supplementary information

Supplementary Information Supplementary Figs. 1–4.

Reporting Summary

## Data Availability

The data that support the findings of this study are available from the corresponding author upon request.

## References

[1] Chugh SS (2014). Worldwide epidemiology of atrial fibrillation: a Global Burden of Disease 2010 Study. Circulation.

[2] Krijthe BP (2013). Projections on the number of individuals with atrial fibrillation in the European Union, from 2000 to 2060. Eur. Heart J..

[3] Schnabel RB (2015). 50 year trends in atrial fibrillation prevalence, incidence, risk factors, and mortality in the Framingham Heart Study: a cohort study. Lancet.

[4] Zoni-Berisso M, Lercari F, Carazza T, Domenicucci S (2014). Epidemiology of atrial fibrillation: European perspective. Clin. Epidemiol.

[5] Bogle, B. M., Ning, H., Mehrotra, S., Goldberger, J. J. & Lloyd-Jones, D. M. Lifetime risk for sudden cardiac death in the community. *J. Am. Heart Assoc.*10.1161/jaha.115.002398 (2016). 10.1161/JAHA.115.002398 PMC5015355 27356557

[6] Kirchhof P (2016). 2016 ESC Guidelines for the management of atrial fibrillation developed in collaboration with EACTS. Eur. Heart J..

[7] Wolf PA, Abbott RD, Kannel WB (1991). Atrial fibrillation as an independent risk factor for stroke: the Framingham Study. Stroke.

[8] Clauss S (2019). Animal models of arrhythmia: classic electrophysiology to genetically modified large animals. Nat. Rev. Cardiol..

[9] Heijman J (2016). The value of basic research insights into atrial fibrillation mechanisms as a guide to therapeutic innovation: a critical analysis. Cardiovasc. Res..

[10] Schüttler D (2020). Animal models of atrial fibrillation. Circ. Res..

[11] Saba S, Wang PJ, Estes NA (2000). 3rd Invasive cardiac electrophysiology in the mouse: techniques and applications. Trends Cardiovasc. Med..

[12] Poon Y-Y (2021). Disproportional cardiovascular depressive effects of isoflurane: serendipitous findings from a comprehensive re-visit in mice. Lab Anim..

[13] Bennett K, Lewis K (2022). Sedation and anesthesia in rodents. Vet. Clin. North Am. Exot. Anim. Pract..

[14] Diven K (2003). Inhalation anesthetics in rodents. Lab Anim..

[15] Grabmaier U (2014). The role of 1.5 Tesla MRI and anesthetic regimen concerning cardiac analysis in mice with cardiomyopathy. PLoS ONE.

[16] Fleischmann T, Jirkof P, Henke J, Arras M, Cesarovic N (2016). Injection anaesthesia with fentanyl-midazolam-medetomidine in adult female mice: importance of antagonization and perioperative care. Lab Anim..

[17] Criado AB, Gómez e Segura IA (2003). Reduction of isoflurane MAC by fentanyl or remifentanil in rats. Vet. Anaesth. Analg..

[18] Hollis AR, Pascal M, Van Dijk J, Jolliffe C, Kaartinen J (2020). Behavioural and cardiovascular effects of medetomidine constant rate infusion compared with detomidine for standing sedation in horses. Vet. Anaesth. Analg..

[19] Perotta JH (2014). Hyoscine-*N*-butylbromide premedication on cardiovascular variables of horses sedated with medetomidine. Vet. Anaesth. Analg..

[20] Kramer S, Nolte I, Jöchle W (1996). Clinical comparison of medetomidine with xylazine/l-methadone in dogs. Vet. Rec..

[21] Alibhai HI, Clarke KW, Lee YH, Thompson J (1996). Cardiopulmonary effects of combinations of medetomidine hydrochloride and atropine sulphate in dogs. Vet. Rec..

[22] Lombard CW, Kvart C, Sateri H, Holm G, Nilsfors L (1989). Effects of medetomidine in dogs with mitral regurgitation. Acta Vet. Scand. Suppl..

[23] Forsyth SF, Machon RG, Walsh VP (1999). Anaesthesia of a Sumatran tiger on eight occasions with ketamine, medetomidine and isoflurane.. NZ Vet. J..

[24] Albrecht M, Henke J, Tacke S, Markert M, Guth B (2014). Effects of isoflurane, ketamine-xylazine and a combination of medetomidine, midazolam and fentanyl on physiological variables continuously measured by telemetry in Wistar rats. BMC Vet. Res..

[25] Tashiro M, Tohei A (2022). Recommended doses of medetomidine–midazolam–butorphanol with atipamezole for preventing hypothermia in mice. J. Vet. Med. Sci..

[26] Cruz JI, Loste JM, Burzaco OH (1998). Observations on the use of medetomidine/ketamine and its reversal with atipamezole for chemical restraint in the mouse. Lab Anim..

[27] Pypendop BH, Verstegen JP (1998). Hemodynamic effects of medetomidine in the dog: a dose titration study. Vet. Surg..

[28] Nakae Y, Kanaya N, Namiki A (1997). The direct effects of diazepam and midazolam on myocardial depression in cultured rat ventricular myocytes. Anesth. Analg..

[29] Brahim JS, Thut PD (1984). Hemodynamic changes during isoflurane anesthesia. Anesth. Prog..

[30] Wolf WJ, Neal MB, Peterson MD (1986). The hemodynamic and cardiovascular effects of isoflurane and halothane anesthesia in children. Anesthesiology.

[31] Wang G (2015). Effects of anesthesia on conventional and speckle tracking echocardiographic parameters in a mouse model of pressure overload. Exp. Ther. Med..

[32] Suzuki A, Aizawa K, Gassmayr S, Bosnjak ZJ, Kwok WM (2002). Biphasic effects of isoflurane on the cardiac action potential: an ionic basis for anesthetic-induced changes in cardiac electrophysiology. Anesthesiology.

[33] Lavoie J (1995). Effects of propofol or isoflurane anesthesia on cardiac conduction in children undergoing radiofrequency catheter ablation for tachydysrhythmias. Anesthesiology.

[34] Appleton GO (2004). Determinants of cardiac electrophysiological properties in mice. J. Interv. Card. Electrophysiol..

[35] Warhol A, George SA, Obaid SN, Efimova T, Efimov IR (2021). Differential cardiotoxic electrocardiographic response to doxorubicin treatment in conscious versus anesthetized mice. Physiol. Rep..

[36] Shintaku T (2014). Effects of propofol on electrocardiogram measures in mice. J. Pharm. Sci..

[37] Bagliani G, Della Rocca DG, Di Biase L, Padeletti L (2017). PR interval and junctional zone. Card. Electrophysiol. Clin..

[38] Cygankiewicz I, Zareba W (2013). Heart rate variability. Handb. Clin. Neurol..

[39] Task Force of the European Society of Cardiology and the North American Society of Pacing and Electrophysiology. Heart rate variability: standards of measurement, physiological interpretation, and clinical use. Eur. Heart J. 17, 354–381 (1996). 8737210

[40] Li P, Sur SH, Mistlberger RE, Morris M (1999). Circadian blood pressure and heart rate rhythms in mice. Am. J. Physiol..

[41] Eger, E. I. 2nd The pharmacology of isoflurane. *Br. J. Anaesth.* **56** (Suppl. 1), 71S–99S (1984). 6391530

[42] Scheinin H, Virtanen R, MacDonald E, Lammintausta R, Scheinin M (1989). Medetomidine—a novel alpha 2-adrenoceptor agonist: a review of its pharmacodynamic effects. Prog. Neuropsychopharmacol. Biol. Psychiatry.

[43] Brennan M, Palaniswami M, Kamen P (2001). Do existing measures of Poincaré plot geometry reflect nonlinear features of heart rate variability?. IEEE Trans. Biomed. Eng..

[44] Roy B, Ghatak S (2013). Nonlinear methods to assess changes in heart rate variability in type 2 diabetic patients. Arq. Bras. Cardiol..

[45] Hayano J, Yuda E (2019). Pitfalls of assessment of autonomic function by heart rate variability. J. Physiol. Anthropol..

[46] Prystowsky EN, Gilge JL (2021). Atrioventricular conduction: physiology and autonomic influences. Card. Electrophysiol. Clin..

[47] Vincentz JW, Rubart M, Firulli AB (2012). Ontogeny of cardiac sympathetic innervation and its implications for cardiac disease. Pediatr. Cardiol..

[48] Randall WC, Milosavljevic M, Wurster RD, Geis GS, Ardell JL (1986). Selective vagal innervation of the heart. Ann. Clin. Lab. Sci..

[49] Freeman LC, Ack JA, Fligner MA, Muir WW (1990). Atrial fibrillation in halothane- and isoflurane-anesthetized dogs. Am. J. Vet. Res..

[50] Kanaya N, Nakayama M, Kobayashi I, Fujita S, Namiki A (1998). Effect of isoflurane on epinephrine-induced arrhythmias in ischemic-reperfused dog hearts. Res. Commun. Mol. Pathol. Pharmacol..

[51] Carbone L, Austin J (2016). Pain and laboratory animals: publication practices for better data reproducibility and better animal welfare. PLoS ONE.

[52] Stokes EL, Flecknell PA, Richardson CA (2009). Reported analgesic and anaesthetic administration to rodents undergoing experimental surgical procedures. Lab Anim..

[53] Navarro KL (2021). Mouse anesthesia: the art and science. ILAR J..

[54] Buitrago S, Martin TE, Tetens-Woodring J, Belicha-Villanueva A, Wilding GE (2008). Safety and efficacy of various combinations of injectable anesthetics in BALB/c mice. J. Am. Assoc. Lab. Anim. Sci..

[55] Rödig G, Keyl C, Wiesner G, Philipp A, Hobbhahn J (1996). Effects of sevoflurane and isoflurane on systemic vascular resistance: use of cardiopulmonary bypass as a study model. Br. J. Anaesth..

[56] Beery AK, Zucker I (2011). Sex bias in neuroscience and biomedical research. Neurosci. Biobehav. Rev..

[57] Soldin OP, Mattison DR (2009). Sex differences in pharmacokinetics and pharmacodynamics. Clin. Pharmacokinet..

[58] Barrett-Connor E (1997). Sex differences in coronary heart disease. Why are women so superior? The 1995 Ancel keys lecture. Circulation.

[59] Klinge I (2008). Gender perspectives in European research. Pharmacol. Res..

[60] Mitchell GF, Jeron A, Koren G (1998). Measurement of heart rate and Q-T interval in the conscious mouse. Am. J. Physiol..

[61] Percie du Sert, N. et al. The ARRIVE guidelines 2.0: updated guidelines for reporting animal research. *PLoS Biol.* **18**, e3000410 (2020). 10.1371/journal.pbio.3000410 PMC7360023 32663219

[62] National Research Council Committee for the Update of the Guide for the Care & Use of Laboratory Animals in *Guide for the Care and Use of Laboratory Animals* (National Academies Press, 2011).

[63] Tomsits, P. et al. Analyzing long-term electrocardiography recordings to detect arrhythmias in mice. *J. Vis. Exp.*10.3791/62386 (2021). 10.3791/62386 34096914

[64] Hulsmans M (2017). Macrophages facilitate electrical conduction in the heart. Cell.

[65] Gehrmann J (2001). Electrophysiological characterization of murine myocardial ischemia and infarction. Basic Res. Cardiol..

[66] Berul CI, Aronovitz MJ, Wang PJ, Mendelsohn ME (1996). In vivo cardiac electrophysiology studies in the mouse. Circulation.

