# Supplementary material for: Medetomidine/midazolam/fentanyl narcosis alters cardiac autonomic tone leading to conduction disorders and arrhythmias in mice
Source: Lab Anim (NY). 2023 Mar 23;52(4):85–92. doi: 10.1038/s41684-023-01141-0 (PMC10063441; doi:10.1038/s41684-023-01141-0)
Supplement: Supplementary file 1 — Supplementary Figs. 1–4. [file 41684_2023_1141_MOESM1_ESM.pdf]

nature portfolio

https://doi.org/10.1038/s41684-023-01141-0

Supplementary information

Medetomidine/midazolam/fentanyl narcosis alters cardiac autonomic tone leading to conduction disorders and arrhythmias in mice

In the format provided by the authors and unedited

Medetomidine/midazolam/fentanyl narcosis alters cardiac autonomic tone, leading to conduction disorders and arrhythmias in mice

Philipp Tomsits[1,2,3*], Lina Volz[1,2,3], Ruibing Xia[1,3], Aparna Chivukula[1,2,3], Dominik Schüttler[1,2,3,5] and Sebastian Clauß[1,2,3,4,5*]

1 Medizinische Klinik und Poliklinik I, University Hospital Munich, Campus Grosshadern and Innenstadt, Ludwig-Maximilians University Munich (LMU), Munich, Germany

2 DZHK (German Centre for Cardiovascular Research), Partner Site Munich, Munich Heart Alliance (MHA), Munich, Germany

3 Institute of Surgical Research at the Walter-Brendel-Centre of Experimental Medicine, University Hospital, LMU Munich, Munich, Germany

4 Interfaculty Center for Endocrine and Cardiovascular Disease Network Modelling and Clinical Transfer (ICONLMU), LMU Munich, Munich, Germany

5 These authors contributed equally to this work

*e-mail: Philipp-Johannes.Tomsits@med.uni-muenchen.de; Sebastian.Clauss@med.uni-muenchen.de

Supplement

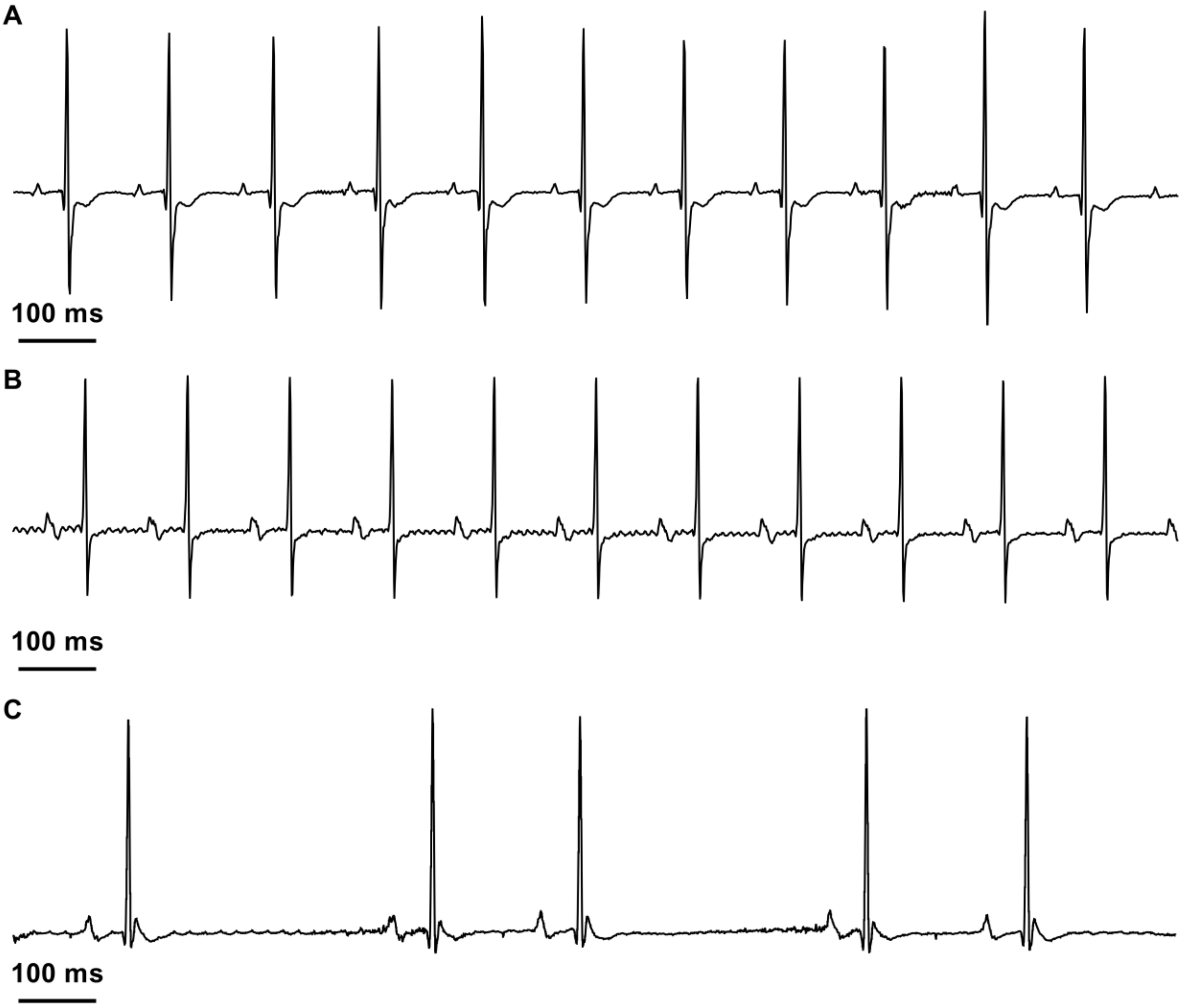

Supplementary Fig. 1 │ Representative ECG traces.

a, Representative ECG obtained by an implanted telemetry device in a lead two configuration from an awake mouse around noon. **b,** Representative ECG obtained by sharp needle electrodes in a lead one configuration right after fully established IF narcosis. **c,** Representative ECG obtained by sharp needle electrodes in a lead one configuration right after fully established MMF narcosis.

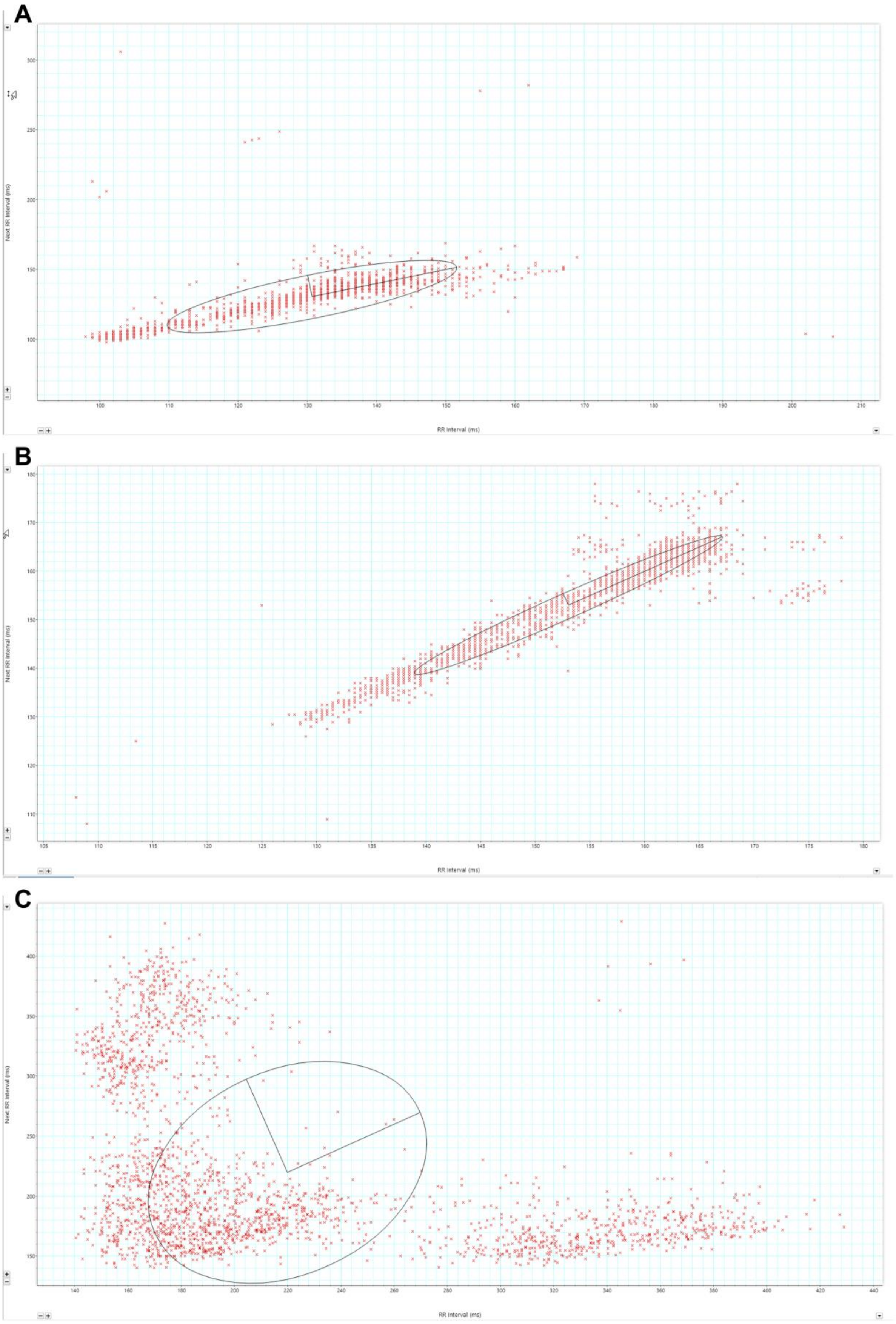

Supplementary Fig. 2 │ Representative Poincaré plots.

a, Representative Poincaré plot obtained by automated HRV analysis using LabChart from an ECG recorded by an implanted telemetry device in a lead II configuration from an awake mouse around noon. **b,** Representative Poincaré plot obtained by automated HRV analysis using LabChart from an ECG recorded by sharp needle electrodes in a lead I configuration right after fully established IF narcosis. **c,** Representative Poincaré plot obtained by automated HRV analysis using LabChart from an ECG recorded by sharp needle electrodes in a lead I configuration right after fully established MMF narcosis.

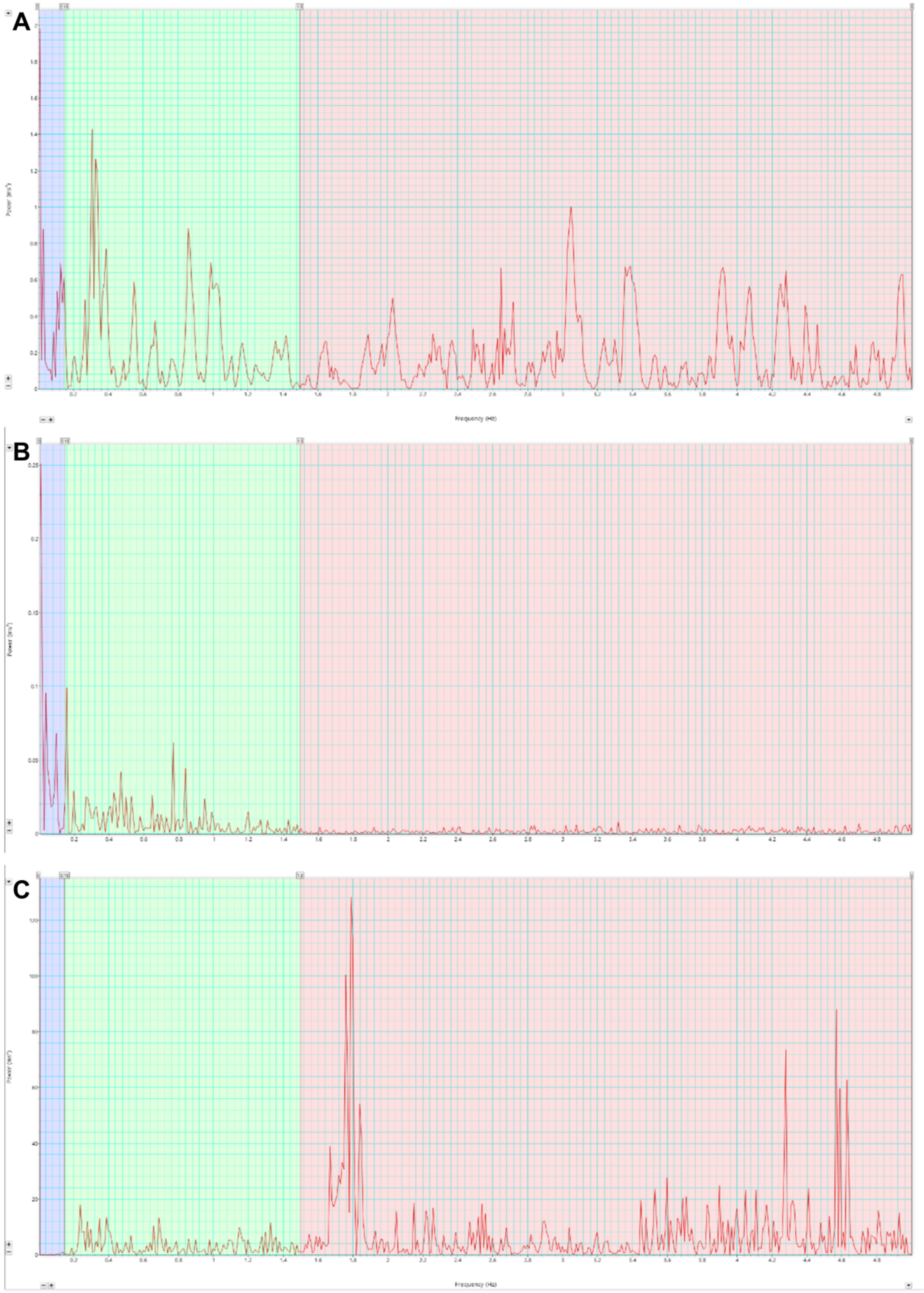

Supplementary Fig. 3 │ Representative Power Spectral Density plots.

a, Representative Power Spectral Density plot obtained by automated HRV analysis using LabChart from an ECG recorded by an implanted telemetry device in a lead II configuration

from an awake mouse around noon. **b,** Representative Power Spectral Density plot obtained by automated HRV analysis using LabChart from an ECG recorded by sharp needle electrodes in a lead I configuration right after fully established IF narcosis. **c,** Representative Power Spectral Density plot obtained by automated HRV analysis using LabChart from an ECG recorded by sharp needle electrodes in a lead I configuration right after fully established MMF narcosis.

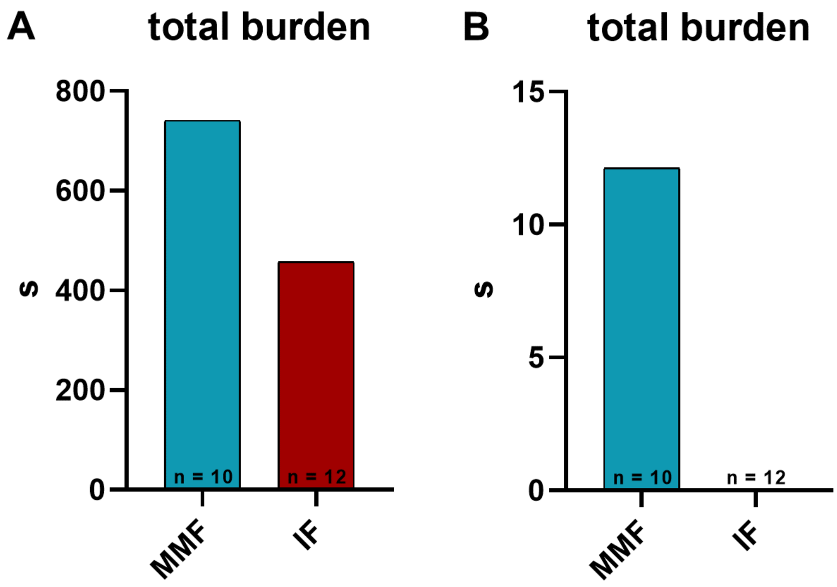

Supplementary Figure 4│ Total Burden.

a, Total atrial arrhythmia burden per group (sum, n=10 for MMF and n=12 for IF respectively). **b,** Total ventricular arrhythmia burden per group (sum, n=12 for MMF and n=10 for IF respectively).
